# Supplementary material for: Reduced intrinsic DNA curvature leads to increased mutation rate
Source: Genome Biol. 2018 Sep 14;19:132. doi: 10.1186/s13059-018-1525-y (PMC6138893; doi:10.1186/s13059-018-1525-y)
Supplement: Supplementary file 1 — Figure S1. Test of the Poisson distribution of nonsense mutations in URA3. Figure S2. The effect of nucleotide at the potential nonsense site on mutation rate. Figure S3. The estimation of the average mutation rate and the values of DNA properties. Figure S4. The results in cancer cells were not affected by SNV calling methods. Figure S5. DNA curvature is negatively associated with mutation rate in coding sequences in human tumors. Figure S6. The results in cancer cells held after controlling for the trinucleotide context. Figure S7. The results in cancer cells held after controlling for the heptanucleotide context. Figure S8. Logistic regression for predicting the presence of a SNV in human tumors. Figure S9. De novo point mutations in the human germline are enriched in DNA regions with a smaller DNA curvature. Figure S10. Expression levels were not significantly different among URA3 variants. Figure S11. Electrophoretic mobility shift assay showing differences in DNA curvature among URA3 variants. Figure S12. Comparison of intrinsic DNA curvature among yeast genes. Figure S13. Comparison of intrinsic DNA curvature among human genes. Figure S14. The distribution of the number of colonies on 60 5-FOA plates for each of the five URA3 variants. Table S1. Numbers of plates containing a mutation in URA3. Table S2. Models on predicting the mutation rate of a potential nonsense site in CAN1. Table S3. Modeling the mutation rate of a potential nonsense site in four human genes. Table S4. DNA sequences of URA3 variants. Table S5. Features of five URA3 variants. Table S6. Primers used in this study. (DOCX 1081 kb) [file 13059_2018_1525_MOESM1_ESM.docx]

**Figure S1.** **Test of the Poisson distribution of nonsense mutations in *URA3*.**

(a) The observed (red arrow) and expected standard deviations (histogram showing 1,000 permutations) of the numbers of nonsense mutations among the potential nonsense mutation sites within the first two-thirds of *URA3* coding sequence. (b-c) The observed and expected standard deviations for transitions (b) and transversions (c), respectively.

**Figure S2. The effect of nucleotide at the potential nonsense site on mutation rate.** Each data point represents a potential nonsense site.

**Figure S3. The estimation of the average mutation rate and the values of DNA properties.** (a) An illustration of the calculation of mutation rate in a region from 251 to 300 (*L* = 50 bp here as an example). Five potential nonsense mutation sites are at 252, 260, 268, 274, and 277. Two of them (274 and 277) are shown in grey bars because mutations were not observed in those sites in our experiment. (b) The values of dinucleotide used for the calculation of the values of DNA properties in this study.

**Figure S4. The results in cancer cells were not affected by SNV calling methods.**

**Figure S5. DNA curvature is negatively associated with mutation rate in coding sequences in human tumors.**

(a) The average intrinsic DNA curvature of SNV-containing regions (red arrows) was significantly smaller than the random expectation in coding sequences (histogram showing 1,000 permutations). *P* value was calculated from a permutation test. (b) The interquartile range of intrinsic DNA curvature of 101 bp DNA sequences randomly sampled from 5′ UTR, coding sequences (CDS), and 3′ UTR. The number of 101 bp DNA sequences sampled from 5′ UTR, CDS, or 3′ UTR was identical to the number of total observed SNVs in the corresponding genomic region in all cancer types. Error bars represent standard errors estimated by bootstrapping. *P* values were calculated from permutation tests.

**Figure S6. The results in cancer cells held after controlling for the trinucleotide context.** That is, in the generation of the random mutation sites, we chose only the pseudo-mutation sites with the same trinucleotide sequence context (the mutation site, +1, and –1 nucleotide) as that for the observed mutation sites.

**Figure S7. The results in cancer cells held after controlling for the heptanucleotide context.**

**Figure S8. Logistic regression for predicting the presence of a SNV in human tumors.**

A logistic regression was performed in *R* with function *glm*. The coordinate indicates the position relative to a SNV site. Positive and negative values indicate downstream and upstream sites, respectively, and 0 indicates the polymorphic site. The coefficient of each categorical variable (such as a change from A to C at the polymorphic site, 0:A>C) in the logistic regression model was used to reflect the effect of the variable. The effect of DNA curvature was estimated from the product of its coefficient in the model and its range observed in the genome. Green and blue bars represent positive and negative effects, respectively.

**Figure S9. *De novo* point mutations in the human germline are enriched in DNA regions with a smaller DNA curvature.** The average intrinsic DNA curvature of SNV-containing regions (red arrows) was significantly smaller than the random expectation (histogram showing 1,000 permutations). *P* value was calculated from a permutation test.

**Figure S10. Expression levels were not significantly different among *URA3* variants.** Expression levels of *URA3* variants were normalized by that of the wild-type. *P* values were calculated using the *t*-test.

**Figure S11. Electrophoretic mobility shift assay showing differences in DNA curvature among *URA3* variants.**

**Figure S12.** **Comparison of intrinsic DNA curvature among yeast genes**. (a) Essential and nonessential genes. (b-g) Genes leading to significantly slow growth upon deletion and those without significant effect on growth rate, in YPD, YP + 5% glycerol, YP + 2% ethanol, SC, mimicked Oak tree extrudate, and YPD + 6% ethanol, respectively. (h) Haploinsufficient and haplosufficient genes. (i) Singleton and duplicate genes. (j) Genes with higher PPI degree (top 50%) and lower PPI degree (bottom 50%). Outliers are not shown. *P* values were calculated from the Mann-Whitney *U* test.

**Figure S13.** **Comparison of intrinsic DNA curvature among human genes**. (a-b) Essential and nonessential genes, the lists of which were retrieved from Hart et al. 2010 and Wang et al. 2015, respectively. (c) Haploinsufficient and haplosufficient genes. (d) Genes with higher PPI degree (top 50%) and lower PPI degree (bottom 50%). The longest open reading frame of each gene was used for the calculation of DNA curvature. Outliers are not shown. *P* values were calculated from the Mann-Whitney *U* test.

**Figure S14. The distribution of the number of colonies on sixty 5-FOA plates for each of the five *URA3* variants.** The variance-to-mean ratios are shown.

**Table S1. Numbers of plates containing a mutation in *URA3*.**

| Position | Wild-type | A | T | G | C |
| --- | --- | --- | --- | --- | --- |
| 1 | A | 0 | 0 | 0 | 0 |
| 2 | T | 0 | 0 | 0 | 0 |
| 3 | G | 0 | 0 | 0 | 0 |
| 4 | T | 0 | 0 | 0 | 0 |
| 5 | C | 0 | 0 | 0 | 0 |
| 6 | G | 0 | 0 | 0 | 0 |
| 7 | A | 0 | 0 | 0 | 0 |
| 8 | A | 0 | 0 | 0 | 0 |
| 9 | A | 0 | 0 | 0 | 0 |
| 10 | G | 0 | 0 | 0 | 0 |
| 11 | C | 0 | 0 | 0 | 0 |
| 12 | T | 0 | 0 | 0 | 0 |
| 13 | A | 0 | 0 | 0 | 0 |
| 14 | C | 0 | 0 | 0 | 0 |
| 15 | A | 0 | 0 | 0 | 0 |
| 16 | T | 0 | 0 | 0 | 0 |
| 17 | A | 0 | 0 | 0 | 0 |
| 18 | T | 1 | 0 | 1 | 0 |
| 19 | A | 0 | 1 | 0 | 0 |
| 20 | A | 0 | 0 | 0 | 0 |
| 21 | G | 0 | 0 | 0 | 0 |
| 22 | G | 0 | 1 | 0 | 0 |
| 23 | A | 0 | 0 | 0 | 0 |
| 24 | A | 0 | 0 | 0 | 0 |
| 25 | C | 0 | 0 | 2 | 0 |
| 26 | G | 2 | 0 | 0 | 2 |
| 27 | T | 0 | 0 | 0 | 0 |
| 28 | G | 0 | 0 | 0 | 1 |
| 29 | C | 0 | 0 | 0 | 0 |
| 30 | T | 0 | 0 | 0 | 0 |
| 31 | G | 1 | 0 | 0 | 0 |
| 32 | C | 0 | 0 | 0 | 0 |
| 33 | T | 0 | 0 | 0 | 0 |
| 34 | A | 0 | 0 | 0 | 0 |
| 35 | C | 0 | 0 | 0 | 0 |
| 36 | T | 0 | 0 | 0 | 0 |
| 37 | C | 0 | 0 | 0 | 0 |
| 38 | A | 0 | 0 | 0 | 0 |
| 39 | T | 0 | 0 | 0 | 0 |
| 40 | C | 0 | 0 | 0 | 0 |
| 41 | C | 0 | 0 | 0 | 0 |
| 42 | T | 0 | 0 | 0 | 0 |
| 43 | A | 0 | 0 | 0 | 0 |
| 44 | G | 0 | 0 | 0 | 0 |
| 45 | T | 0 | 0 | 0 | 0 |
| 46 | C | 0 | 0 | 0 | 0 |
| 47 | C | 0 | 0 | 0 | 0 |
| 48 | T | 0 | 0 | 0 | 0 |
| 49 | G | 0 | 0 | 0 | 0 |
| 50 | T | 1 | 0 | 0 | 0 |
| 51 | T | 0 | 0 | 0 | 0 |
| 52 | G | 0 | 0 | 0 | 0 |
| 53 | C | 0 | 0 | 0 | 0 |
| 54 | T | 0 | 0 | 0 | 0 |
| 55 | G | 0 | 0 | 0 | 0 |
| 56 | C | 0 | 0 | 0 | 0 |
| 57 | C | 0 | 0 | 0 | 0 |
| 58 | A | 0 | 0 | 0 | 0 |
| 59 | A | 0 | 0 | 0 | 0 |
| 60 | G | 0 | 0 | 0 | 0 |
| 61 | C | 0 | 0 | 0 | 0 |
| 62 | T | 0 | 0 | 0 | 2 |
| 63 | A | 0 | 0 | 0 | 0 |
| 64 | T | 0 | 0 | 0 | 0 |
| 65 | T | 0 | 0 | 0 | 0 |
| 66 | T | 0 | 0 | 0 | 0 |
| 67 | A | 0 | 0 | 0 | 0 |
| 68 | A | 0 | 0 | 0 | 0 |
| 69 | T | 0 | 0 | 0 | 0 |
| 70 | A | 0 | 0 | 0 | 0 |
| 71 | T | 0 | 0 | 0 | 1 |
| 72 | C | 1 | 0 | 0 | 0 |
| 73 | A | 0 | 1 | 0 | 0 |
| 74 | T | 0 | 0 | 1 | 0 |
| 75 | G | 0 | 0 | 0 | 0 |
| 76 | C | 0 | 0 | 0 | 0 |
| 77 | A | 0 | 0 | 0 | 1 |
| 78 | C | 0 | 0 | 0 | 0 |
| 79 | G | 0 | 3 | 0 | 0 |
| 80 | A | 0 | 0 | 0 | 0 |
| 81 | A | 0 | 0 | 0 | 0 |
| 82 | A | 0 | 1 | 0 | 0 |
| 83 | A | 0 | 0 | 0 | 0 |
| 84 | G | 0 | 0 | 0 | 0 |
| 85 | C | 0 | 2 | 0 | 0 |
| 86 | A | 0 | 0 | 0 | 0 |
| 87 | A | 0 | 0 | 0 | 0 |
| 88 | A | 0 | 0 | 0 | 0 |
| 89 | C | 1 | 2 | 0 | 0 |
| 90 | A | 0 | 0 | 0 | 0 |
| 91 | A | 0 | 0 | 0 | 0 |
| 92 | A | 0 | 0 | 0 | 0 |
| 93 | C | 5 | 0 | 1 | 0 |
| 94 | T | 0 | 0 | 0 | 0 |
| 95 | T | 0 | 0 | 0 | 4 |
| 96 | G | 0 | 0 | 0 | 0 |
| 97 | T | 0 | 0 | 0 | 1 |
| 98 | G | 3 | 1 | 0 | 0 |
| 99 | T | 1 | 0 | 0 | 0 |
| 100 | G | 0 | 0 | 0 | 0 |
| 101 | C | 1 | 0 | 0 | 0 |
| 102 | T | 0 | 0 | 0 | 0 |
| 103 | T | 0 | 0 | 0 | 0 |
| 104 | C | 0 | 2 | 0 | 0 |
| 105 | A | 0 | 0 | 0 | 0 |
| 106 | T | 0 | 0 | 0 | 0 |
| 107 | T | 0 | 0 | 0 | 0 |
| 108 | G | 0 | 0 | 0 | 0 |
| 109 | G | 0 | 0 | 0 | 0 |
| 110 | A | 0 | 0 | 0 | 0 |
| 111 | T | 0 | 0 | 0 | 0 |
| 112 | G | 0 | 0 | 0 | 0 |
| 113 | T | 0 | 0 | 0 | 0 |
| 114 | T | 0 | 0 | 0 | 0 |
| 115 | C | 0 | 0 | 0 | 0 |
| 116 | G | 0 | 0 | 0 | 0 |
| 117 | T | 0 | 0 | 0 | 0 |
| 118 | A | 0 | 0 | 0 | 0 |
| 119 | C | 0 | 0 | 0 | 0 |
| 120 | C | 0 | 0 | 0 | 0 |
| 121 | A | 0 | 0 | 0 | 0 |
| 122 | C | 0 | 0 | 0 | 0 |
| 123 | C | 0 | 0 | 0 | 0 |
| 124 | A | 0 | 0 | 0 | 0 |
| 125 | A | 0 | 0 | 0 | 0 |
| 126 | G | 0 | 0 | 0 | 0 |
| 127 | G | 0 | 1 | 0 | 0 |
| 128 | A | 0 | 0 | 0 | 0 |
| 129 | A | 0 | 0 | 0 | 0 |
| 130 | T | 0 | 0 | 0 | 0 |
| 131 | T | 0 | 0 | 0 | 0 |
| 132 | A | 0 | 0 | 0 | 0 |
| 133 | C | 0 | 0 | 0 | 0 |
| 134 | T | 0 | 0 | 0 | 2 |
| 135 | G | 0 | 0 | 0 | 0 |
| 136 | G | 0 | 2 | 0 | 0 |
| 137 | A | 0 | 0 | 0 | 0 |
| 138 | G | 0 | 0 | 0 | 0 |
| 139 | T | 0 | 0 | 0 | 0 |
| 140 | T | 0 | 0 | 1 | 0 |
| 141 | A | 0 | 0 | 0 | 0 |
| 142 | G | 0 | 1 | 0 | 0 |
| 143 | T | 1 | 0 | 0 | 0 |
| 144 | T | 0 | 0 | 0 | 0 |
| 145 | G | 0 | 1 | 0 | 0 |
| 146 | A | 0 | 0 | 0 | 0 |
| 147 | A | 0 | 0 | 0 | 0 |
| 148 | G | 0 | 0 | 0 | 0 |
| 149 | C | 0 | 0 | 0 | 0 |
| 150 | A | 0 | 0 | 0 | 0 |
| 151 | T | 0 | 0 | 0 | 0 |
| 152 | T | 1 | 0 | 2 | 0 |
| 153 | A | 0 | 0 | 0 | 0 |
| 154 | G | 0 | 0 | 0 | 0 |
| 155 | G | 3 | 0 | 0 | 0 |
| 156 | T | 0 | 0 | 0 | 0 |
| 157 | C | 0 | 0 | 0 | 0 |
| 158 | C | 0 | 0 | 0 | 0 |
| 159 | C | 0 | 0 | 0 | 0 |
| 160 | A | 0 | 1 | 0 | 0 |
| 161 | A | 0 | 0 | 0 | 0 |
| 162 | A | 0 | 0 | 0 | 0 |
| 163 | A | 0 | 0 | 0 | 0 |
| 164 | T | 1 | 0 | 0 | 0 |
| 165 | T | 0 | 0 | 0 | 0 |
| 166 | T | 0 | 0 | 0 | 0 |
| 167 | G | 6 | 6 | 0 | 0 |
| 168 | T | 2 | 0 | 1 | 0 |
| 169 | T | 0 | 0 | 0 | 0 |
| 170 | T | 2 | 0 | 0 | 0 |
| 171 | A | 0 | 0 | 0 | 0 |
| 172 | C | 0 | 0 | 0 | 0 |
| 173 | T | 0 | 0 | 0 | 0 |
| 174 | A | 0 | 0 | 0 | 0 |
| 175 | A | 0 | 0 | 0 | 0 |
| 176 | A | 0 | 1 | 0 | 2 |
| 177 | A | 0 | 0 | 0 | 1 |
| 178 | A | 0 | 0 | 0 | 0 |
| 179 | C | 2 | 0 | 0 | 0 |
| 180 | A | 0 | 0 | 0 | 0 |
| 181 | C | 0 | 3 | 0 | 0 |
| 182 | A | 0 | 1 | 0 | 1 |
| 183 | T | 0 | 0 | 0 | 0 |
| 184 | G | 0 | 0 | 0 | 0 |
| 185 | T | 0 | 0 | 0 | 0 |
| 186 | G | 0 | 0 | 0 | 0 |
| 187 | G | 0 | 0 | 0 | 0 |
| 188 | A | 0 | 0 | 0 | 0 |
| 189 | T | 0 | 0 | 0 | 0 |
| 190 | A | 0 | 0 | 0 | 0 |
| 191 | T | 0 | 0 | 0 | 0 |
| 192 | C | 0 | 0 | 0 | 0 |
| 193 | T | 0 | 0 | 0 | 0 |
| 194 | T | 4 | 0 | 0 | 0 |
| 195 | G | 0 | 0 | 0 | 0 |
| 196 | A | 0 | 0 | 0 | 0 |
| 197 | C | 0 | 0 | 0 | 0 |
| 198 | T | 0 | 0 | 0 | 0 |
| 199 | G | 0 | 1 | 0 | 0 |
| 200 | A | 0 | 0 | 0 | 0 |
| 201 | T | 0 | 0 | 0 | 0 |
| 202 | T | 0 | 0 | 0 | 0 |
| 203 | T | 0 | 0 | 0 | 0 |
| 204 | T | 0 | 0 | 0 | 0 |
| 205 | T | 0 | 0 | 0 | 0 |
| 206 | C | 0 | 0 | 0 | 0 |
| 207 | C | 0 | 0 | 0 | 0 |
| 208 | A | 0 | 0 | 0 | 0 |
| 209 | T | 0 | 0 | 0 | 0 |
| 210 | G | 0 | 0 | 0 | 0 |
| 211 | G | 0 | 3 | 0 | 0 |
| 212 | A | 0 | 0 | 0 | 0 |
| 213 | G | 0 | 0 | 0 | 0 |
| 214 | G | 0 | 0 | 0 | 0 |
| 215 | G | 0 | 0 | 0 | 0 |
| 216 | C | 0 | 0 | 0 | 0 |
| 217 | A | 0 | 0 | 0 | 1 |
| 218 | C | 1 | 0 | 2 | 0 |
| 219 | A | 0 | 0 | 0 | 0 |
| 220 | G | 0 | 0 | 0 | 0 |
| 221 | T | 1 | 0 | 0 | 0 |
| 222 | T | 0 | 0 | 0 | 0 |
| 223 | A | 0 | 1 | 0 | 0 |
| 224 | A | 0 | 0 | 0 | 0 |
| 225 | G | 0 | 0 | 0 | 0 |
| 226 | C | 0 | 0 | 0 | 0 |
| 227 | C | 0 | 0 | 0 | 0 |
| 228 | G | 0 | 0 | 0 | 0 |
| 229 | C | 0 | 0 | 0 | 0 |
| 230 | T | 1 | 0 | 1 | 1 |
| 231 | A | 0 | 0 | 0 | 0 |
| 232 | A | 0 | 0 | 0 | 0 |
| 233 | A | 0 | 0 | 0 | 0 |
| 234 | G | 0 | 0 | 0 | 0 |
| 235 | G | 0 | 0 | 0 | 0 |
| 236 | C | 0 | 0 | 0 | 0 |
| 237 | A | 0 | 0 | 0 | 0 |
| 238 | T | 0 | 0 | 0 | 0 |
| 239 | T | 0 | 0 | 0 | 0 |
| 240 | A | 0 | 0 | 0 | 0 |
| 241 | T | 0 | 0 | 0 | 5 |
| 242 | C | 0 | 3 | 0 | 0 |
| 243 | C | 0 | 0 | 0 | 0 |
| 244 | G | 0 | 0 | 0 | 0 |
| 245 | C | 0 | 0 | 0 | 0 |
| 246 | C | 0 | 0 | 0 | 0 |
| 247 | A | 0 | 0 | 0 | 0 |
| 248 | A | 0 | 0 | 0 | 0 |
| 249 | G | 0 | 0 | 0 | 0 |
| 250 | T | 0 | 0 | 0 | 0 |
| 251 | A | 0 | 0 | 0 | 0 |
| 252 | C | 1 | 0 | 1 | 0 |
| 253 | A | 0 | 0 | 0 | 0 |
| 254 | A | 0 | 0 | 0 | 0 |
| 255 | T | 0 | 0 | 0 | 0 |
| 256 | T | 0 | 0 | 0 | 0 |
| 257 | T | 0 | 0 | 0 | 2 |
| 258 | T | 0 | 0 | 0 | 0 |
| 259 | T | 0 | 0 | 0 | 0 |
| 260 | T | 2 | 0 | 0 | 0 |
| 261 | A | 0 | 0 | 0 | 0 |
| 262 | C | 0 | 0 | 0 | 0 |
| 263 | T | 0 | 0 | 0 | 0 |
| 264 | C | 0 | 0 | 0 | 0 |
| 265 | T | 0 | 0 | 0 | 0 |
| 266 | T | 0 | 0 | 0 | 0 |
| 267 | C | 0 | 0 | 0 | 0 |
| 268 | G | 9 | 4 | 0 | 0 |
| 269 | A | 0 | 0 | 0 | 0 |
| 270 | A | 0 | 0 | 0 | 0 |
| 271 | G | 6 | 2 | 0 | 0 |
| 272 | A | 0 | 2 | 0 | 1 |
| 273 | C | 0 | 0 | 2 | 0 |
| 274 | A | 0 | 0 | 0 | 0 |
| 275 | G | 0 | 1 | 0 | 0 |
| 276 | A | 0 | 0 | 0 | 0 |
| 277 | A | 0 | 0 | 0 | 2 |
| 278 | A | 0 | 5 | 3 | 0 |
| 279 | A | 0 | 0 | 0 | 2 |
| 280 | T | 0 | 0 | 0 | 0 |
| 281 | T | 0 | 0 | 0 | 0 |
| 282 | T | 0 | 0 | 0 | 0 |
| 283 | G | 0 | 0 | 0 | 3 |
| 284 | C | 0 | 0 | 0 | 0 |
| 285 | T | 0 | 0 | 0 | 0 |
| 286 | G | 2 | 2 | 0 | 1 |
| 287 | A | 0 | 1 | 0 | 3 |
| 288 | C | 0 | 0 | 0 | 0 |
| 289 | A | 0 | 0 | 0 | 0 |
| 290 | T | 0 | 0 | 0 | 0 |
| 291 | T | 0 | 0 | 0 | 0 |
| 292 | G | 0 | 0 | 0 | 0 |
| 293 | G | 0 | 0 | 0 | 0 |
| 294 | T | 0 | 0 | 0 | 0 |
| 295 | A | 0 | 0 | 0 | 0 |
| 296 | A | 0 | 0 | 0 | 0 |
| 297 | T | 0 | 0 | 0 | 0 |
| 298 | A | 0 | 0 | 0 | 0 |
| 299 | C | 0 | 0 | 0 | 0 |
| 300 | A | 0 | 0 | 0 | 0 |
| 301 | G | 0 | 0 | 0 | 0 |
| 302 | T | 0 | 0 | 0 | 0 |
| 303 | C | 0 | 0 | 0 | 0 |
| 304 | A | 0 | 0 | 0 | 0 |
| 305 | A | 0 | 0 | 0 | 0 |
| 306 | A | 0 | 0 | 0 | 0 |
| 307 | T | 0 | 0 | 0 | 0 |
| 308 | T | 0 | 0 | 0 | 0 |
| 309 | G | 0 | 0 | 0 | 0 |
| 310 | C | 0 | 6 | 0 | 0 |
| 311 | A | 0 | 0 | 0 | 1 |
| 312 | G | 0 | 0 | 0 | 1 |
| 313 | T | 1 | 0 | 0 | 0 |
| 314 | A | 0 | 0 | 0 | 0 |
| 315 | C | 1 | 0 | 1 | 0 |
| 316 | T | 0 | 0 | 0 | 0 |
| 317 | C | 0 | 0 | 0 | 0 |
| 318 | T | 0 | 0 | 0 | 0 |
| 319 | G | 0 | 0 | 0 | 1 |
| 320 | C | 0 | 0 | 0 | 0 |
| 321 | G | 0 | 0 | 0 | 0 |
| 322 | G | 0 | 0 | 0 | 0 |
| 323 | G | 0 | 0 | 0 | 0 |
| 324 | T | 0 | 0 | 0 | 0 |
| 325 | G | 0 | 0 | 0 | 0 |
| 326 | T | 0 | 0 | 0 | 0 |
| 327 | A | 0 | 0 | 0 | 0 |
| 328 | T | 0 | 0 | 0 | 0 |
| 329 | A | 0 | 0 | 0 | 0 |
| 330 | C | 4 | 0 | 3 | 0 |
| 331 | A | 0 | 0 | 0 | 0 |
| 332 | G | 0 | 0 | 0 | 0 |
| 333 | A | 0 | 0 | 0 | 0 |
| 334 | A | 0 | 0 | 0 | 0 |
| 335 | T | 0 | 0 | 0 | 0 |
| 336 | A | 0 | 0 | 0 | 0 |
| 337 | G | 0 | 0 | 0 | 0 |
| 338 | C | 3 | 0 | 0 | 0 |
| 339 | A | 0 | 0 | 0 | 0 |
| 340 | G | 0 | 4 | 0 | 0 |
| 341 | A | 0 | 0 | 0 | 0 |
| 342 | A | 0 | 0 | 0 | 0 |
| 343 | T | 1 | 0 | 1 | 0 |
| 344 | G | 8 | 3 | 0 | 0 |
| 345 | G | 3 | 4 | 0 | 0 |
| 346 | G | 0 | 0 | 0 | 0 |
| 347 | C | 2 | 0 | 0 | 0 |
| 348 | A | 0 | 0 | 0 | 0 |
| 349 | G | 0 | 0 | 0 | 0 |
| 350 | A | 0 | 0 | 0 | 0 |
| 351 | C | 0 | 0 | 0 | 0 |
| 352 | A | 0 | 0 | 0 | 0 |
| 353 | T | 1 | 0 | 0 | 0 |
| 354 | T | 0 | 0 | 0 | 0 |
| 355 | A | 0 | 0 | 0 | 1 |
| 356 | C | 4 | 0 | 2 | 0 |
| 357 | G | 0 | 0 | 0 | 0 |
| 358 | A | 0 | 0 | 0 | 1 |
| 359 | A | 0 | 0 | 0 | 0 |
| 360 | T | 0 | 0 | 3 | 0 |
| 361 | G | 0 | 0 | 0 | 0 |
| 362 | C | 5 | 0 | 0 | 0 |
| 363 | A | 0 | 0 | 0 | 0 |
| 364 | C | 0 | 0 | 0 | 0 |
| 365 | A | 0 | 0 | 0 | 0 |
| 366 | C | 0 | 0 | 0 | 0 |
| 367 | G | 0 | 0 | 0 | 0 |
| 368 | G | 2 | 0 | 0 | 0 |
| 369 | T | 0 | 0 | 0 | 0 |
| 370 | G | 0 | 1 | 0 | 0 |
| 371 | T | 1 | 0 | 0 | 0 |
| 372 | G | 0 | 0 | 0 | 0 |
| 373 | G | 0 | 0 | 0 | 0 |
| 374 | T | 0 | 0 | 0 | 0 |
| 375 | G | 0 | 0 | 0 | 0 |
| 376 | G | 0 | 0 | 0 | 1 |
| 377 | G | 0 | 1 | 0 | 0 |
| 378 | C | 0 | 0 | 0 | 0 |
| 379 | C | 0 | 0 | 0 | 0 |
| 380 | C | 0 | 1 | 0 | 0 |
| 381 | A | 0 | 0 | 0 | 0 |
| 382 | G | 0 | 0 | 0 | 0 |
| 383 | G | 0 | 0 | 0 | 0 |
| 384 | T | 0 | 0 | 0 | 0 |
| 385 | A | 0 | 1 | 0 | 0 |
| 386 | T | 0 | 0 | 0 | 0 |
| 387 | T | 0 | 0 | 0 | 0 |
| 388 | G | 0 | 0 | 0 | 0 |
| 389 | T | 0 | 0 | 0 | 0 |
| 390 | T | 0 | 0 | 0 | 0 |
| 391 | A | 0 | 0 | 0 | 0 |
| 392 | G | 0 | 0 | 0 | 0 |
| 393 | C | 0 | 0 | 0 | 0 |
| 394 | G | 0 | 0 | 0 | 0 |
| 395 | G | 0 | 0 | 0 | 0 |
| 396 | T | 0 | 0 | 0 | 0 |
| 397 | T | 0 | 0 | 0 | 0 |
| 398 | T | 0 | 0 | 2 | 1 |
| 399 | G | 0 | 0 | 0 | 0 |
| 400 | A | 0 | 0 | 0 | 0 |
| 401 | A | 0 | 0 | 0 | 0 |
| 402 | G | 0 | 0 | 0 | 0 |
| 403 | C | 0 | 1 | 0 | 0 |
| 404 | A | 0 | 0 | 0 | 0 |
| 405 | G | 0 | 0 | 0 | 0 |
| 406 | G | 0 | 0 | 0 | 0 |
| 407 | C | 0 | 0 | 0 | 0 |
| 408 | G | 0 | 0 | 0 | 0 |
| 409 | G | 0 | 0 | 0 | 0 |
| 410 | C | 0 | 0 | 0 | 0 |
| 411 | G | 0 | 0 | 0 | 0 |
| 412 | G | 0 | 1 | 0 | 0 |
| 413 | A | 0 | 0 | 0 | 0 |
| 414 | A | 0 | 0 | 0 | 0 |
| 415 | G | 0 | 0 | 0 | 0 |
| 416 | A | 0 | 0 | 0 | 0 |
| 417 | A | 0 | 0 | 0 | 0 |
| 418 | G | 0 | 0 | 0 | 0 |
| 419 | T | 0 | 0 | 0 | 0 |
| 420 | A | 0 | 0 | 0 | 0 |
| 421 | A | 0 | 0 | 0 | 0 |
| 422 | C | 0 | 0 | 0 | 0 |
| 423 | A | 0 | 0 | 0 | 0 |
| 424 | A | 0 | 0 | 0 | 0 |
| 425 | A | 0 | 0 | 0 | 0 |
| 426 | G | 0 | 0 | 0 | 0 |
| 427 | G | 0 | 2 | 0 | 0 |
| 428 | A | 0 | 0 | 0 | 0 |
| 429 | A | 0 | 0 | 0 | 0 |
| 430 | C | 0 | 0 | 0 | 0 |
| 431 | C | 0 | 0 | 0 | 0 |
| 432 | T | 0 | 0 | 0 | 0 |
| 433 | A | 0 | 0 | 1 | 0 |
| 434 | G | 0 | 1 | 0 | 0 |
| 435 | A | 0 | 3 | 0 | 2 |
| 436 | G | 0 | 0 | 0 | 5 |
| 437 | G | 3 | 0 | 0 | 0 |
| 438 | C | 0 | 0 | 0 | 0 |
| 439 | C | 0 | 0 | 0 | 0 |
| 440 | T | 2 | 0 | 0 | 0 |
| 441 | T | 0 | 0 | 0 | 0 |
| 442 | T | 0 | 0 | 0 | 0 |
| 443 | T | 0 | 0 | 0 | 0 |
| 444 | G | 0 | 0 | 0 | 0 |
| 445 | A | 0 | 0 | 0 | 0 |
| 446 | T | 1 | 0 | 0 | 0 |
| 447 | G | 0 | 0 | 0 | 0 |
| 448 | T | 0 | 0 | 0 | 0 |
| 449 | T | 1 | 0 | 2 | 0 |
| 450 | A | 0 | 0 | 0 | 0 |
| 451 | G | 0 | 0 | 0 | 0 |
| 452 | C | 0 | 0 | 0 | 0 |
| 453 | A | 0 | 0 | 0 | 0 |
| 454 | G | 0 | 3 | 0 | 0 |
| 455 | A | 0 | 0 | 0 | 0 |
| 456 | A | 0 | 0 | 0 | 0 |
| 457 | T | 0 | 0 | 0 | 0 |
| 458 | T | 0 | 0 | 0 | 0 |
| 459 | G | 0 | 0 | 0 | 0 |
| 460 | T | 0 | 0 | 0 | 0 |
| 461 | C | 0 | 0 | 2 | 0 |
| 462 | A | 0 | 0 | 0 | 0 |
| 463 | T | 0 | 0 | 0 | 0 |
| 464 | G | 0 | 0 | 0 | 0 |
| 465 | C | 1 | 0 | 0 | 0 |
| 466 | A | 0 | 0 | 0 | 0 |
| 467 | A | 0 | 0 | 0 | 0 |
| 468 | G | 0 | 0 | 0 | 0 |
| 469 | G | 0 | 0 | 0 | 0 |
| 470 | G | 0 | 0 | 0 | 0 |
| 471 | C | 0 | 0 | 0 | 0 |
| 472 | T | 0 | 0 | 0 | 0 |
| 473 | C | 0 | 0 | 0 | 0 |
| 474 | C | 0 | 0 | 0 | 0 |
| 475 | C | 0 | 0 | 0 | 0 |
| 476 | T | 0 | 0 | 0 | 0 |
| 477 | A | 0 | 0 | 0 | 0 |
| 478 | T | 0 | 0 | 0 | 0 |
| 479 | C | 0 | 0 | 0 | 0 |
| 480 | T | 0 | 0 | 0 | 0 |
| 481 | A | 0 | 0 | 0 | 0 |
| 482 | C | 0 | 0 | 0 | 0 |
| 483 | T | 0 | 0 | 0 | 0 |
| 484 | G | 0 | 1 | 0 | 0 |
| 485 | G | 0 | 0 | 0 | 0 |
| 486 | A | 0 | 0 | 0 | 0 |
| 487 | G | 0 | 0 | 0 | 0 |
| 488 | A | 0 | 0 | 0 | 0 |
| 489 | A | 0 | 0 | 0 | 0 |
| 490 | T | 0 | 0 | 0 | 0 |
| 491 | A | 0 | 0 | 0 | 0 |
| 492 | T | 1 | 0 | 1 | 0 |
| 493 | A | 0 | 0 | 0 | 0 |
| 494 | C | 0 | 0 | 0 | 0 |
| 495 | T | 0 | 0 | 0 | 0 |
| 496 | A | 0 | 0 | 0 | 0 |
| 497 | A | 0 | 0 | 0 | 0 |
| 498 | G | 0 | 0 | 0 | 0 |
| 499 | G | 0 | 0 | 0 | 0 |
| 500 | G | 0 | 0 | 0 | 0 |
| 501 | T | 0 | 0 | 0 | 0 |
| 502 | A | 0 | 0 | 0 | 0 |
| 503 | C | 0 | 0 | 0 | 0 |
| 504 | T | 0 | 0 | 0 | 0 |
| 505 | G | 0 | 0 | 0 | 0 |
| 506 | T | 0 | 0 | 0 | 0 |
| 507 | T | 0 | 0 | 0 | 0 |
| 508 | G | 0 | 0 | 0 | 0 |
| 509 | A | 0 | 0 | 0 | 0 |
| 510 | C | 0 | 0 | 0 | 0 |
| 511 | A | 0 | 0 | 0 | 0 |
| 512 | T | 0 | 0 | 0 | 0 |
| 513 | T | 0 | 0 | 0 | 0 |
| 514 | G | 0 | 0 | 0 | 1 |
| 515 | C | 2 | 0 | 0 | 0 |
| 516 | G | 0 | 0 | 0 | 0 |
| 517 | A | 0 | 0 | 0 | 0 |
| 518 | A | 0 | 0 | 0 | 0 |
| 519 | G | 0 | 0 | 0 | 0 |
| 520 | A | 0 | 0 | 0 | 0 |
| 521 | G | 0 | 0 | 0 | 0 |
| 522 | C | 0 | 0 | 0 | 0 |
| 523 | G | 0 | 0 | 0 | 0 |
| 524 | A | 0 | 0 | 0 | 0 |
| 525 | C | 0 | 0 | 0 | 0 |
| 526 | A | 0 | 1 | 0 | 0 |
| 527 | A | 0 | 0 | 0 | 0 |
| 528 | A | 0 | 0 | 0 | 0 |
| 529 | G | 0 | 0 | 0 | 0 |
| 530 | A | 0 | 0 | 0 | 0 |
| 531 | T | 0 | 0 | 0 | 0 |
| 532 | T | 0 | 0 | 0 | 0 |
| 533 | T | 0 | 0 | 0 | 0 |
| 534 | T | 0 | 0 | 0 | 0 |
| 535 | G | 0 | 3 | 0 | 0 |
| 536 | T | 2 | 0 | 0 | 0 |
| 537 | T | 0 | 0 | 0 | 0 |
| 538 | A | 0 | 0 | 0 | 0 |
| 539 | T | 0 | 0 | 0 | 0 |
| 540 | C | 0 | 0 | 0 | 0 |
| 541 | G | 9 | 0 | 0 | 0 |
| 542 | G | 2 | 2 | 0 | 2 |
| 543 | C | 0 | 0 | 0 | 0 |
| 544 | T | 0 | 0 | 0 | 0 |
| 545 | T | 0 | 0 | 0 | 2 |
| 546 | T | 0 | 0 | 0 | 0 |
| 547 | A | 0 | 0 | 0 | 0 |
| 548 | T | 0 | 0 | 0 | 0 |
| 549 | T | 0 | 0 | 0 | 0 |
| 550 | G | 0 | 0 | 0 | 2 |
| 551 | C | 0 | 0 | 0 | 0 |
| 552 | T | 1 | 0 | 0 | 0 |
| 553 | C | 0 | 3 | 0 | 0 |
| 554 | A | 0 | 0 | 0 | 0 |
| 555 | A | 0 | 0 | 0 | 0 |
| 556 | A | 0 | 0 | 0 | 0 |
| 557 | G | 0 | 0 | 0 | 0 |
| 558 | A | 0 | 0 | 0 | 0 |
| 559 | G | 0 | 0 | 0 | 0 |
| 560 | A | 0 | 0 | 0 | 0 |
| 561 | C | 0 | 0 | 0 | 0 |
| 562 | A | 0 | 0 | 0 | 0 |
| 563 | T | 0 | 0 | 0 | 0 |
| 564 | G | 0 | 0 | 0 | 0 |
| 565 | G | 0 | 0 | 0 | 0 |
| 566 | G | 0 | 0 | 0 | 0 |
| 567 | T | 0 | 0 | 0 | 0 |
| 568 | G | 0 | 2 | 0 | 0 |
| 569 | G | 0 | 0 | 0 | 0 |
| 570 | A | 0 | 0 | 0 | 0 |
| 571 | A | 0 | 0 | 0 | 0 |
| 572 | G | 0 | 0 | 0 | 0 |
| 573 | A | 0 | 0 | 0 | 0 |
| 574 | G | 0 | 0 | 0 | 0 |
| 575 | A | 0 | 0 | 0 | 0 |
| 576 | T | 0 | 0 | 0 | 0 |
| 577 | G | 0 | 1 | 0 | 0 |
| 578 | A | 0 | 0 | 0 | 0 |
| 579 | A | 0 | 0 | 0 | 0 |
| 580 | G | 0 | 0 | 0 | 0 |
| 581 | G | 0 | 0 | 0 | 0 |
| 582 | T | 0 | 0 | 0 | 0 |
| 583 | T | 0 | 0 | 0 | 0 |
| 584 | A | 0 | 0 | 0 | 0 |
| 585 | C | 7 | 0 | 1 | 0 |
| 586 | G | 0 | 0 | 0 | 0 |
| 587 | A | 0 | 0 | 0 | 0 |
| 588 | T | 0 | 0 | 0 | 0 |
| 589 | T | 0 | 0 | 0 | 0 |
| 590 | G | 7 | 0 | 0 | 0 |
| 591 | G | 5 | 0 | 0 | 0 |
| 592 | T | 0 | 0 | 0 | 0 |
| 593 | T | 0 | 0 | 0 | 0 |
| 594 | G | 0 | 0 | 0 | 0 |
| 595 | A | 0 | 0 | 0 | 0 |
| 596 | T | 0 | 0 | 0 | 0 |
| 597 | T | 0 | 0 | 0 | 0 |
| 598 | A | 0 | 0 | 0 | 0 |
| 599 | T | 0 | 0 | 1 | 0 |
| 600 | G | 0 | 0 | 0 | 0 |
| 601 | A | 0 | 0 | 0 | 2 |
| 602 | C | 2 | 0 | 0 | 0 |
| 603 | A | 0 | 0 | 0 | 0 |
| 604 | C | 0 | 0 | 0 | 0 |
| 605 | C | 4 | 4 | 1 | 0 |
| 606 | C | 0 | 1 | 0 | 0 |
| 607 | G | 0 | 0 | 0 | 0 |
| 608 | G | 7 | 1 | 0 | 0 |
| 609 | T | 0 | 0 | 0 | 0 |
| 610 | G | 0 | 0 | 0 | 0 |
| 611 | T | 0 | 0 | 0 | 0 |
| 612 | G | 0 | 0 | 0 | 0 |
| 613 | G | 0 | 0 | 0 | 0 |
| 614 | G | 0 | 0 | 0 | 0 |
| 615 | T | 0 | 0 | 0 | 0 |
| 616 | T | 0 | 0 | 0 | 0 |
| 617 | T | 0 | 0 | 0 | 0 |
| 618 | A | 0 | 0 | 0 | 0 |
| 619 | G | 0 | 0 | 0 | 0 |
| 620 | A | 0 | 0 | 0 | 0 |
| 621 | T | 0 | 0 | 0 | 0 |
| 622 | G | 0 | 0 | 0 | 0 |
| 623 | A | 0 | 0 | 0 | 0 |
| 624 | C | 0 | 0 | 0 | 0 |
| 625 | A | 0 | 0 | 0 | 0 |
| 626 | A | 0 | 0 | 0 | 0 |
| 627 | G | 0 | 0 | 0 | 0 |
| 628 | G | 0 | 2 | 0 | 0 |
| 629 | G | 0 | 0 | 0 | 0 |
| 630 | A | 0 | 0 | 0 | 0 |
| 631 | G | 0 | 0 | 0 | 0 |
| 632 | A | 0 | 0 | 0 | 0 |
| 633 | C | 0 | 0 | 0 | 0 |
| 634 | G | 0 | 0 | 0 | 0 |
| 635 | C | 0 | 0 | 0 | 0 |
| 636 | A | 0 | 0 | 0 | 0 |
| 637 | T | 0 | 0 | 0 | 0 |
| 638 | T | 0 | 0 | 0 | 0 |
| 639 | G | 0 | 0 | 0 | 0 |
| 640 | G | 0 | 0 | 0 | 0 |
| 641 | G | 0 | 0 | 0 | 0 |
| 642 | T | 0 | 0 | 0 | 0 |
| 643 | C | 0 | 6 | 0 | 0 |
| 644 | A | 0 | 0 | 0 | 0 |
| 645 | A | 0 | 0 | 0 | 0 |
| 646 | C | 0 | 0 | 0 | 0 |
| 647 | A | 0 | 0 | 0 | 0 |
| 648 | G | 0 | 0 | 0 | 0 |
| 649 | T | 0 | 0 | 0 | 0 |
| 650 | A | 0 | 0 | 0 | 0 |
| 651 | T | 1 | 0 | 3 | 0 |
| 652 | A | 0 | 1 | 0 | 0 |
| 653 | G | 0 | 0 | 0 | 0 |
| 654 | A | 0 | 0 | 0 | 0 |
| 655 | A | 0 | 0 | 0 | 0 |
| 656 | C | 0 | 0 | 0 | 0 |
| 657 | C | 0 | 0 | 0 | 0 |
| 658 | G | 0 | 0 | 0 | 0 |
| 659 | T | 0 | 0 | 0 | 0 |
| 660 | G | 0 | 0 | 0 | 0 |
| 661 | G | 0 | 0 | 0 | 0 |
| 662 | A | 0 | 0 | 0 | 0 |
| 663 | T | 0 | 0 | 0 | 0 |
| 664 | G | 0 | 0 | 0 | 0 |
| 665 | A | 0 | 0 | 0 | 0 |
| 666 | T | 0 | 0 | 0 | 0 |
| 667 | G | 0 | 0 | 0 | 0 |
| 668 | T | 0 | 0 | 0 | 0 |
| 669 | G | 0 | 0 | 0 | 0 |
| 670 | G | 0 | 0 | 0 | 0 |
| 671 | T | 0 | 0 | 0 | 0 |
| 672 | C | 0 | 0 | 0 | 0 |
| 673 | T | 0 | 0 | 0 | 0 |
| 674 | C | 0 | 0 | 0 | 0 |
| 675 | T | 0 | 0 | 0 | 0 |
| 676 | A | 0 | 0 | 0 | 0 |
| 677 | C | 0 | 0 | 0 | 0 |
| 678 | A | 0 | 0 | 0 | 0 |
| 679 | G | 0 | 2 | 0 | 0 |
| 680 | G | 0 | 0 | 0 | 0 |
| 681 | A | 0 | 0 | 0 | 0 |
| 682 | T | 0 | 0 | 0 | 0 |
| 683 | C | 3 | 2 | 0 | 0 |
| 684 | T | 0 | 0 | 0 | 0 |
| 685 | G | 1 | 3 | 0 | 1 |
| 686 | A | 0 | 3 | 0 | 0 |
| 687 | C | 0 | 0 | 0 | 0 |
| 688 | A | 0 | 0 | 0 | 0 |
| 689 | T | 0 | 0 | 0 | 0 |
| 690 | T | 0 | 0 | 0 | 0 |
| 691 | A | 0 | 0 | 0 | 0 |
| 692 | T | 1 | 0 | 0 | 0 |
| 693 | T | 0 | 0 | 0 | 0 |
| 694 | A | 0 | 0 | 0 | 0 |
| 695 | T | 0 | 0 | 0 | 0 |
| 696 | T | 0 | 0 | 0 | 0 |
| 697 | G | 0 | 7 | 0 | 0 |
| 698 | T | 2 | 0 | 0 | 0 |
| 699 | T | 0 | 0 | 0 | 0 |
| 700 | G | 4 | 2 | 0 | 1 |
| 701 | G | 1 | 1 | 0 | 0 |
| 702 | A | 0 | 0 | 0 | 0 |
| 703 | A | 0 | 1 | 0 | 0 |
| 704 | G | 0 | 0 | 0 | 0 |
| 705 | A | 0 | 0 | 0 | 0 |
| 706 | G | 0 | 2 | 0 | 0 |
| 707 | G | 0 | 0 | 0 | 0 |
| 708 | A | 0 | 0 | 0 | 0 |
| 709 | C | 0 | 0 | 0 | 0 |
| 710 | T | 0 | 0 | 1 | 0 |
| 711 | A | 0 | 0 | 0 | 0 |
| 712 | T | 0 | 0 | 0 | 0 |
| 713 | T | 0 | 0 | 0 | 0 |
| 714 | T | 0 | 0 | 0 | 0 |
| 715 | G | 0 | 0 | 0 | 0 |
| 716 | C | 0 | 0 | 0 | 0 |
| 717 | A | 0 | 0 | 0 | 0 |
| 718 | A | 0 | 0 | 0 | 0 |
| 719 | A | 0 | 0 | 0 | 0 |
| 720 | G | 0 | 0 | 0 | 0 |
| 721 | G | 0 | 0 | 0 | 0 |
| 722 | G | 0 | 0 | 0 | 0 |
| 723 | A | 0 | 0 | 0 | 0 |
| 724 | A | 0 | 0 | 0 | 0 |
| 725 | G | 0 | 0 | 0 | 0 |
| 726 | G | 0 | 0 | 0 | 0 |
| 727 | G | 0 | 0 | 0 | 0 |
| 728 | A | 0 | 1 | 0 | 0 |
| 729 | T | 0 | 0 | 0 | 0 |
| 730 | G | 0 | 0 | 0 | 0 |
| 731 | C | 0 | 0 | 0 | 0 |
| 732 | T | 0 | 0 | 0 | 0 |
| 733 | A | 0 | 1 | 0 | 0 |
| 734 | A | 0 | 0 | 0 | 0 |
| 735 | G | 1 | 0 | 0 | 0 |
| 736 | G | 0 | 0 | 0 | 0 |
| 737 | T | 0 | 0 | 0 | 0 |
| 738 | A | 0 | 0 | 0 | 0 |
| 739 | G | 0 | 0 | 0 | 0 |
| 740 | A | 0 | 0 | 0 | 0 |
| 741 | G | 0 | 0 | 0 | 0 |
| 742 | G | 0 | 0 | 0 | 0 |
| 743 | G | 0 | 0 | 0 | 0 |
| 744 | T | 0 | 0 | 0 | 0 |
| 745 | G | 0 | 0 | 0 | 0 |
| 746 | A | 0 | 0 | 0 | 0 |
| 747 | A | 0 | 0 | 0 | 0 |
| 748 | C | 0 | 0 | 0 | 0 |
| 749 | G | 0 | 0 | 0 | 0 |
| 750 | T | 1 | 0 | 0 | 0 |
| 751 | T | 0 | 0 | 0 | 0 |
| 752 | A | 0 | 0 | 0 | 1 |
| 753 | C | 2 | 0 | 0 | 0 |
| 754 | A | 0 | 0 | 0 | 0 |
| 755 | G | 0 | 1 | 0 | 0 |
| 756 | A | 0 | 0 | 0 | 0 |
| 757 | A | 0 | 1 | 0 | 0 |
| 758 | A | 0 | 0 | 0 | 0 |
| 759 | A | 0 | 0 | 0 | 0 |
| 760 | G | 0 | 0 | 0 | 0 |
| 761 | C | 0 | 0 | 0 | 0 |
| 762 | A | 0 | 0 | 0 | 0 |
| 763 | G | 0 | 0 | 0 | 2 |
| 764 | G | 6 | 1 | 0 | 0 |
| 765 | C | 0 | 0 | 0 | 0 |
| 766 | T | 0 | 0 | 3 | 0 |
| 767 | G | 6 | 0 | 0 | 0 |
| 768 | G | 7 | 0 | 0 | 0 |
| 769 | G | 0 | 2 | 0 | 0 |
| 770 | A | 0 | 0 | 0 | 0 |
| 771 | A | 0 | 0 | 0 | 0 |
| 772 | G | 0 | 0 | 0 | 0 |
| 773 | C | 0 | 0 | 0 | 0 |
| 774 | A | 0 | 0 | 0 | 0 |
| 775 | T | 0 | 0 | 0 | 0 |
| 776 | A | 0 | 0 | 0 | 0 |
| 777 | T | 2 | 0 | 0 | 0 |
| 778 | T | 0 | 0 | 0 | 0 |
| 779 | T | 0 | 0 | 0 | 0 |
| 780 | G | 0 | 0 | 0 | 0 |
| 781 | A | 0 | 0 | 0 | 0 |
| 782 | G | 0 | 0 | 0 | 0 |
| 783 | A | 0 | 0 | 0 | 0 |
| 784 | A | 0 | 0 | 0 | 0 |
| 785 | G | 0 | 0 | 0 | 0 |
| 786 | A | 0 | 0 | 0 | 0 |
| 787 | T | 0 | 0 | 0 | 0 |
| 788 | G | 0 | 0 | 0 | 0 |
| 789 | C | 0 | 0 | 0 | 0 |
| 790 | G | 0 | 0 | 0 | 0 |
| 791 | G | 0 | 0 | 0 | 0 |
| 792 | C | 0 | 0 | 0 | 0 |
| 793 | C | 0 | 0 | 0 | 0 |
| 794 | A | 0 | 0 | 0 | 0 |
| 795 | G | 0 | 0 | 0 | 0 |
| 796 | C | 0 | 0 | 0 | 0 |
| 797 | A | 0 | 0 | 0 | 0 |
| 798 | A | 0 | 0 | 0 | 0 |
| 799 | A | 0 | 0 | 0 | 0 |
| 800 | A | 0 | 0 | 0 | 0 |
| 801 | C | 0 | 0 | 0 | 0 |
| 802 | T | 0 | 0 | 0 | 0 |
| 803 | A | 0 | 0 | 0 | 0 |
| 804 | A | 0 | 0 | 0 | 0 |

**Table S2. Models on predicting the mutation rate of a potential nonsense site in *CAN1***

|  | Model | AIC |
| --- | --- | --- |
| 1 | Null model | 389 |
| 2 | Mutation rate ~ “0” ^*^ | 361 |
| 3 | Mutation rate ~ “0” + “+1” + “–1” | 369 |
| 4 | Mutation rate ~ “0” + “+1” + “+2” +“+3” + “–1”+ “–2” +“–3” | 372 |
| 5 | Mutation rate ~ curvature ^**^ | 389 |
| 6 | Mutation rate ~ “0” + curvature | 360 |

^*^ “0” represents the nucleotide at the potential nonsense site. “+1” and “–1” represent the downstream and the upstream nucleotide of the potential nonsense site, respectively.

^**^ The intrinsic DNA curvature in a 101 bp region from 50 bp upstream to 50 bp downstream of the potential nonsense site.

**Table S3. Modelling the mutation rate of a potential nonsense site in 4 human genes**

| Gene Symbol | AIC of Model 1^*^ | AIC of Model 2^**^ | *P* value^***^ |
| --- | --- | --- | --- |
| *TP53* | 445 | 441 | 0.01 |
| *MECP2* | 94 | 91 | 0.01 |
| *NF1* | 101 | 100 | 0.03 |
| *RB1* | 39 | 36 | 0.01 |

^*^ Model 1: Null model (the formula used in *R*): log(mutation rate) ~ 1. When mutation rate at a site is equal to 0, it is artificially assigned to 0.5 to avoid infinite numbers in the log-transformation.

^**^ Model 2: log(mutation rate) ~ curvature. The intrinsic DNA curvature was calculated in a 101 bp region from 50 bp upstream to 50 bp downstream of a potential nonsense site.

^***^ One-tailed *P* value for the coefficient of intrinsic DNA curvature in Model 2. The alternative hypothesis: intrinsic DNA curvature represses mutation.

**Table S4. DNA sequences of *URA3* variants.**

|  | DNA sequence (5′-3′) |
| --- | --- |
| G1 | ATGTCGAAAGCTACTTATAAGGAGAGAGCTGCTACTCATCCTTCTCCTGTTGCTGCTAAGCTTTTTAATATCATGCACGAAAAGCAAACAAATCTTTGCGCTTCTCTTGATGTTAGAACTACTAAGGAACTTCTTGAGCTTGTTGAAGCTCTTGGTCCTAAAATTTGCCTTCTAAAAACTCATGTTGATATTTTGACTGATTTTTCTATGGAGGGAACTGTTAAGCCTCTAAAGGCTCTTTCTGCTAAGTACAATTTTCTTCTTTTCGAAGACAGAAAATTTGCTGACATTGGAAATACAGTCAAACTTCAGTATTCTGCTGGAGTTTACAGAATTGCAGAATGGGCAGACATTACGAATGCTCACGGAGTTGTTGGCCCAGGAATTGTTTCTGGTTTGAAGCAGGCTGCTGAAGAAGTAACAAAGGAGCCTAGAGGCCTTTTGATGCTTGCAGAACTTTCTTGCAAGGGCTCTCTTGCTACTGGAGAATATACTAAGGGAACTGTTGACATTGCGAAGAGCGACAAAGATTTTGTTATCGGCTTTATTGCTCAAAGAGACATGGGAGGAAGAGATGAAGGTTACGATTGGCTTATTATGACTCCTGGAGTTGGTCTTGATGACAAGGGAGACGCTCTTGGTCAGCAGTATAGAACTGTTGATGATGTTGTTTCTACAGGATCTGACATTATTATTGTTGGAAGAGGACTTTTTGCAAAGGGAAGAGATGCTAAGGTTGAGGGAGAGAGATACAGAAAAGCAGGCTGGGAAGCTTATTTGAGAAGATGCGGCCAGCAAAATTAA |
| G2 | ATGTCGAAAGCTACATATAAGGAACGTGCTGCTACTCATCCTAGTCCTGTTGCTGCCAAGCTTTTTAATATCATGCACGAAAAGCAAACAAATCTTTGCGCTTCTCTTGATGTTAGAACTACTAAGGAACTTCTTGAGCTTGTTGAAGCTCTTGGTCCTAAAATTTGCCTTCTAAAAACTCATGTGGATATCTTGACTGATTTTTCCATGGAGGGCACAGTTAAGCCGCTAAAGGCATTATCTGCTAAGTACAATTTTCTTCTTTTCGAAGACAGAAAATTTGCTGACATTGGAAATACAGTCAAACTTCAGTATTCTGCTGGAGTTTACAGAATTGCAGAATGGGCAGACATTACGAATGCTCACGGAGTTGTTGGCCCAGGAATTGTTTCTGGTTTGAAGCAGGCTGCTGAAGAAGTAACAAAGGAGCCTAGAGGCCTTTTGATGCTTGCAGAACTTTCTTGCAAGGGCTCTCTTGCTACTGGAGAATATACTAAGGGTACTGTTGACATTGCGAAGAGCGACAAAGATTTTGTTATCGGCTTTATTGCTCAAAGAGACATGGGAGGAAGAGATGAAGGTTACGATTGGCTTATTATGACTCCTGGAGTTGGTCTTGATGACAAGGGAGACGCTCTTGGTCAGCAGTATAGAACTGTTGATGATGTTGTTTCTACAGGATCTGACATTATTATTGTTGGAAGAGGACTTTTTGCAAAGGGAAGAGATGCTAAGGTTGAGGGAGAGAGATACAGAAAAGCAGGCTGGGAAGCTTATTTGAGAAGATGCGGCCAGCAAAATTAA |
| S1 | ATGTCCAAAGCCACATATAAGGAACGTGCCGCCACCCACCCCTCCCCCGTAGCCGCCAAACTATTTAATATAATGCACGAAAAACAAACCAACTTATGTGCCTCATTGGATGTACGTACCACCAAGGAATTACTGGAGTTAGTAGAAGCATTAGGTCCCAAAATATGTTTACTAAAAACCCATGTGGATATATTAACCGACTTCTCCATGGAGGGTACCGTTAAACCCCTAAAGGCATTATCCGCCAAGTACAACTTCTTATTATTCGAAGACCGTAAATTTGCCGACATAGGTAATACCGTAAAATTACAGTACTCCGCGGGTGTATACCGTATAGCCGAATGGGCCGACATTACCAATGCCCACGGTGTGGTGGGTCCCGGTATAGTTAGCGGTTTAAAACAGGCGGCGGAAGAAGTAACCAAGGAACCCCGTGGTTTATTAATGTTAGCCGAATTATCATGTAAGGGTTCCCTAGCCACCGGTGAATATACCAAGGGTACCGTAGACATAGCCAAGTCCGACAAAGACTTTGTTATAGGTTTTATAGCCCAACGTGACATGGGTGGTCGTGATGAAGGTTACGACTGGTTAATTATGACCCCCGGTGTGGGTTTAGATGACAAGGGTGACGCATTGGGTCAACAGTATCGTACCGTGGATGATGTGGTATCCACCGGATCCGACATTATTATAGTAGGTCGTGGACTATTTGCCAAGGGTAGGGATGCCAAGGTAGAGGGTGAACGTTACCGTAAAGCCGGTTGGGAAGCATACTTACGTAGATGTGGTCAACAAAACTAA |
| S2 | ATGTCCAAAGCCACATATAAGGAACGTGCCGCCACCCACCCCTCCCCCGTAGCCGCCAAACTATTTAATATAATGCACGAAAAACAAACCAACTTATGTGCCTCATTGGATGTACGTACCACCAAGGAATTACTGGAGTTAGTTGAAGCATTAGGTCCCAAAATTTGTTTACTAAAAACACATGTGGATATATTAACCGACTTCTCCATGGAGGGTACCGTTAAACCCCTAAAGGCATTATCCGCCAAGTACAACTTCTTATTATTCGAAGACCGTAAATTTGCCGACATAGGTAATACCGTAAAATTACAGTACTCCGCGGGTGTATACCGTATAGCCGAATGGGCCGACATTACCAATGCCCACGGTGTGGTGGGTCCCGGTATAGTTAGCGGTTTAAAACAGGCGGCGGAAGAAGTAACCAAGGAACCCCGTGGTTTATTAATGTTAGCCGAATTATCATGTAAGGGTTCCCTAGCCACCGGTGAATATACCAAGGGTACCGTAGACATAGCCAAGTCCGACAAAGACTTTGTTATAGGTTTTATAGCCCAACGTGACATGGGTGGTCGTGATGAAGGTTACGACTGGTTAATTATGACACCCGGTGTGGGTTTAGATGACAAGGGAGACGCATTGGGTCAACAGTATCGTACCGTGGATGATGTGGTCTCTACAGGATCTGACATTATTATTGTTGGAAGAGGACTATTTGCAAAGGGTAGGGATGCCAAGGTAGAGGGTGAACGTTACCGTAAAGCAGGCTGGGAAGCATACTTACGTAGATGTGGTCAACAAAACTAA |

**Table S5. Features of five *URA3* variants.**

| Variant | Intrinsic DNA curvature | MFE | GC content | CAI |
| --- | --- | --- | --- | --- |
| WT | 4.48 | -0.99 | 43% | 0.18 |
| G1 | 5.06 | -1.25 | 41% | 0.18 |
| G2 | 4.95 | -1.24 | 42% | 0.17 |
| S1 | 3.88 | -1.45 | 46% | 0.18 |
| S2 | 3.98 | -1.33 | 45% | 0.18 |

**Table S6. Primers used in this study.**

| Primer | DNA sequence (5′-3′) |
| --- | --- |
| *URA3*-amplification-F | TGCGAGGCATATTTATGGTGAA |
| *URA3*-amplification-R | ATAAAGGCCATGAAGCTTTTTCTT |
| *URA3*-PAGE-F | AGATAAATCATGTCCAAAGC |
| *URA3*-PAGE-R | ACTTATAATACAGTTTTTTA |
| *URA3*-qPCR-F | ACCATCAAAGAAGGTTAATGTGGC |
| *URA3*-qPCR-R | GTTTCTTCAACACCACATATGCGTA |
| *ACT1*-qPCR-F | CTCCTCGTGCTGTCTTCCCAT |
| *ACT1*-qPCR-R | CAGATCTTTTCCATATCGTCCCAG |
| *msh2*::natMX-F | TCAACTGTAAAAAATCTCTTTATCTGCTGACCTAACATCAAAATCCTCAGATTAAAAGTAGATCTGTTTAGCTTGCCTT |
| *msh2*::natMX-R | ACTATTTGTATCTATATATTATCTATCGATTCTCACTTAAGATGTCGTTGTAATATTAAATCGATGAATTCGAGCTCGT |
