# Supplementary material for: Reduced intrinsic DNA curvature leads to increased mutation rate
Source: Genome Biol. 2018 Sep 14;19:132. doi: 10.1186/s13059-018-1525-y (PMC6138893; doi:10.1186/s13059-018-1525-y)
Supplement: Supplementary file 2 — Review history. (DOCX 41 kb) [file 13059_2018_1525_MOESM2_ESM.docx]

Review History

**First round of review**

**Reviewer 1:**

**Are the methods appropriate to the aims of the study, are they well described, and are necessary controls included?**
Yes, but could be expanded.

**Are the conclusions adequately supported by the data shown?**

yes assuming that stats tests will be expanded

**Are sufficient details provided to allow replication and comparison with related analyses that may have been performed?**

yes

**Does the work represent a significant advance over previously published studies? Please explain.**

Not sure

**Is the paper of broad interest to others in the field, or of outstanding interest to a broad audience of biologists?**

No

Comments to authors:

Overall, I think this is a good paper which studied the relationship between intrinsic DNA curvature and mutation rate, both in correlation manner and in causality manner, taking into consideration various biological aspects. 

A short summary:
The mutation rate is measured by counting the number of plates containing nonsense mutation (normalized by expected number), the intrinsic DNA curvature is calculated by averaging the parameters obtained from upstream and downstream dinucleotides. 
When using linear models to determine whether DNA curvature is a significant factor correlated with mutation rate, the author also taken into consideration the confounding factors such as GC content, and nucleosome occupancy (respectively: not correlated with mutation rate/ after controlled for, still see significant correlation between DNA curvature and mutation rate). 
After obtaining statistically significant correlation between intrinsic DNA curvature and mutation rate by studying a specific gene in yeast, the authors also confirmed their idea on a genome-wide basis in yeast (experiment using mutation accumulation assay) and human tumors (computationally, comparing intrinsic curvature around SNP with curvature around randomly sampled locus as background). 
Following that, the authors further constructed mutation sequences to experimentally validate the causal relationship (When designing the mutation sequence, the authors kept other factors that might influence mutation rate largely unchanged, such as GC content and codon usage. They also ran an electrophoretic mobility shift assay to confirm the alternation of DNA curvature before conducting the experiment). 
In the next two sections, the authors tried to dissect the biological mechanism behind the phenomenon seen. Through a knockout experiment, they ruled out the possibility that the net mutation rate change observed is caused by increased mismatch repair efficacy, consolidated their claim that the altered net mutation rate by DNA curvature is due to the modulation of supply of mutation. Secondly, by studying the different types of mutation in cancer cells, the authors demonstrated computationally that mutation types that are sensitive to mutagens is more affected by DNA curvature, and brought up the hypothesis that DNA curvature may promote protein binding that makes DNA less accessible to mutagens. 
Finally, the authors stated their study's significance in understanding the evolutionary trajectory, since their work studied the mutation landscape, complimentary to the previous studies focusing on the fitness landscape. 

Some strength of this paper: 
1. The statistical tests used in this study are mainly permutation tests (non-parametric), which construct the background distribution based on random sampling without any excessive non-realistic assumptions. 
2. in their modeling/mutation sequence design, they take into consideration various other factors that might confound the result, such as GC content, nucleosome occupancy, and codon usage. 
3. the biological significance is well demonstrated, by extending their study into human cancer cell mutation.
4. the biological insight/ mechanism behind the result is well elaborated, both by experimental knockout to rule out the effect of mismatch repair efficacy, and by computationally study the relationship between mutagen associated specific type of mutation in cancer cells.

Some suggestions:
1. In the section where the authors extend the study to human cancer cell mutations, the background distribution was obtained by random sampling of locus in the whole genome. This make sense because the authors wanted to study on the genome-wide scale. But I was thinking, additionally, the authors can also conduct a study where they restrict the SNPs they study in certain genomic regions (such as promotor), and obtain the background by also only sampling from this type of genomic region, there might be more interesting results that could be detected. 
2. Maybe the title of the paper could be reconsidered? Something more specific, such as "reduced intrinsic DNA curvature leads to decreased mutation rate"?
3. For the subtitle of the second last section ("DNA curvature reduces mutagen sensitivity in cancer cells"), I find it a little bit too definitive and conclusive, since this section is mainly putting forward this hypothesis, rather than showing the causality through experimental result.

**Reviewer 2:**

**Are the methods appropriate to the aims of the study, are they well described, and are necessary controls included?**
Yes

**Are the conclusions adequately supported by the data shown?**

yes

**Are sufficient details provided to allow replication and comparison with related analyses that may have been performed?**

yes

**Does the work represent a significant advance over previously published studies? Please explain.**

yes

**Is the paper of broad interest to others in the field, or of outstanding interest to a broad audience of biologists?**

Yes: Understanding the process of mutation is important and this manuscript describes a significant advance

Comments to author:

Comments on Duan et al. "DNA local structure decreases mutation rates"

In this interesting manuscript the authors investigate the distribution of new nonsense mutations in two genes URA3 and CAN1. In both cases they find that nonsense mutations are unevenly distributed across sites. They find that the factor which explains this distribution best is the curvature of the DNA; more curved sections have lower mutation rates. They show that this pattern is also evident in human cancer genomes; somatic mutations tend to be found in regions with lower curvature. They test whether the pattern is due to curvature by engineering genes in yeast with more or less curvature and showing that increasing curvature decreases the mutation rate. Also, using a repair deficient strain they show that the relationship between curvature and the mutation rate persists, and hence conclude that the correlation between curvature and the mutation rate is not driven by DNA repair, but is due to curved DNA being less accessible to mutagens.

These are interesting results and the analysis and experiments are appropriate. The effect in yeast seems to be stronger than in humans, with curvature seeming to affect the mutation rate by several fold, whereas curvature only affects certain mutational types in cancers, and not as strongly. Do the authors have any explanation for this? Given the rather modest effects in human cancer genomes I wonder whether curvature is a major determinant of the mutation rate in human somatic tissue. Some insight might be gained by performing logistic regression (sites either have a de novo mutation or not) with curvature as one of many variables in the model. I would accept that this might be beyond the scope of the current project.

Smith et al. (2016 Human Mutation) have found, just as the current authors have, that nonsense mutations are unevenly distributed across sites within human genes. It would be very interesting to see whether this pattern can be explained by curvature.

It would also be interesting to see whether curvature explains some of the variation in the rate of de novo mutation in the human germline; there are now over 140,000 DNMs that have been detected in trios. Again this is probably work for the future.

It is my standard policy to sign my reviews

Adam Eyre-Walker

**Authors’ response to reviews:**

Comments from Editors

As you will see from the reports, both referees are broadly favorable and find the work of potential interest, but they raise important issues that we must ask you to address, in the form of a revised manuscript, before we reach a final decision on publication. In particular, it seems to us to be essential that you address Referee 2's suggestion regarding the difference observed between yeast and human cancer cells when investigating the impact of curvature on the mutation rate and to perform logistic regression. We also ask you to assess the data from Smith et al. to see whether the uneven distribution of the nonsense mutations could be explained by DNA curvature as suggested by Referee 2. As suggested by Referee 1, please conduct a study where you restrict the SNPs in certain genomic regions and obtain the background by only sampling from this type of regions. Please ensure that these and all other issues raised by the referees are addressed in full.

*Response to Editors*

*We would like to take this opportunity to thank the editors and reviewers for the time committed in reviewing our manuscript and for their insightful comments. We have revised the manuscript accordingly. Specifically, we performed logistic regression in human cancer cells, explained the difference in the impact of curvature on mutation rate between yeast and human cells, assessed the data from Smith et al., and performed the permutation test as suggested by Reviewer #1. All new results strengthen the conclusions of our manuscript. We hope that the revised manuscript is suitable for publication in Genome Biology.*

Point-by-point response to Reviewer #1

Comment #1

Overall, I think this is a good paper which studied the relationship between intrinsic DNA curvature and mutation rate, both in correlation manner and in causality manner, taking into consideration various biological aspects.

A short summary:

The mutation rate is measured by counting the number of plates containing nonsense mutation (normalized by expected number), the intrinsic DNA curvature is calculated by averaging the parameters obtained from upstream and downstream dinucleotides. When using linear models to determine whether DNA curvature is a significant factor correlated with mutation rate, the author also taken into consideration the confounding factors such as GC content, and nucleosome occupancy (respectively: not correlated with mutation rate/ after controlled for, still see significant correlation between DNA curvature and mutation rate). After obtaining statistically significant correlation between intrinsic DNA curvature and mutation rate by studying a specific gene in yeast, the authors also confirmed their idea on a genome-wide basis in yeast (experiment using mutation accumulation assay) and human tumors (computationally, comparing intrinsic curvature around SNP with curvature around randomly sampled locus as background). Following that, the authors further constructed mutation sequences to experimentally validate the causal relationship (When designing the mutation sequence, the authors kept other factors that might influence mutation rate largely unchanged, such as GC content and codon usage. They also ran an electrophoretic mobility shift assay to confirm the alternation of DNA curvature before conducting the experiment). In the next two sections, the authors tried to dissect the biological mechanism behind the phenomenon seen. Through a knockout experiment, they ruled out the possibility that the net mutation rate change observed is caused by increased mismatch repair efficacy, consolidated their claim that the altered net mutation rate by DNA curvature is due to the modulation of supply of mutation. Secondly, by studying the different types of mutation in cancer cells, the authors demonstrated computationally that mutation types that are sensitive to mutagens is more affected by DNA curvature, and brought up the hypothesis that DNA curvature may promote protein binding that makes DNA less accessible to mutagens. Finally, the authors stated their study's significance in understanding the evolutionary trajectory, since their work studied the mutation landscape, complimentary to the previous studies focusing on the fitness landscape.

Some strength of this paper:

1. The statistical tests used in this study are mainly permutation tests (non-parametric), which construct the background distribution based on random sampling without any excessive non-realistic assumptions.

2. in their modeling/mutation sequence design, they take into consideration various other factors that might confound the result, such as GC content, nucleosome occupancy, and codon usage.

3. the biological significance is well demonstrated, by extending their study into human cancer cell mutation.

4. the biological insight/ mechanism behind the result is well elaborated, both by experimental knockout to rule out the effect of mismatch repair efficacy, and by computationally study the relationship between mutagen associated specific type of mutation in cancer cells.

*Response #1*

*We thank Reviewer #1 for the positive evaluation.*

Comment #2

Some suggestions:

1. In the section where the authors extend the study to human cancer cell mutations, the background distribution was obtained by random sampling of locus in the whole genome. This make sense because the authors wanted to study on the genome-wide scale. But I was thinking, additionally, the authors can also conduct a study where they restrict the SNPs they study in certain genomic regions (such as promotor), and obtain the background by also only sampling from this type of genomic region, there might be more interesting results that could be detected.

*Response #2*

*We thank Reviewer #1 for the valuable suggestion. Following this suggestion, we separated SNPs into different genomic regions. Because the boundary of 5′ UTR, coding sequences, and 3′ UTR are well annotated, we performed permutation tests for SNPs in each of these three regions. We obtained the expectation by only sampling sequences from the corresponding genomic region. Intriguingly, DNA curvature was negatively associated with mutation rate only in coding sequences. This is possibly related to the broadest range of DNA curvature in coding sequences among these three genomic regions, because it is easier to observe correlations if variables span a broader range. It is also possible that unique mechanisms exist in coding sequences, which requires further investigation in the future. We added a supplementary figure (Figure S5) to show these results in the revised manuscript.*

Comment #3

2. Maybe the title of the paper could be reconsidered? Something more specific, such as "reduced intrinsic DNA curvature leads to decreased mutation rate"?

*Response #3*

*We agree. We changed the title to “Reduced intrinsic DNA curvature leads to increased mutation rate”.*

Comment #4

3. For the subtitle of the second last section ("DNA curvature reduces mutagen sensitivity in cancer cells"), I find it a little bit too definitive and conclusive, since this section is mainly putting forward this hypothesis, rather than showing the causality through experimental result.

*Response #4*

*We agree. We changed the subtitle to “DNA curvature is negatively correlated with mutagen sensitivity in human cancer cells”.*

Point-by-point response to Reviewer #2

Comment #1

Comments on Duan et al. "DNA local structure decreases mutation rates"

In this interesting manuscript the authors investigate the distribution of new nonsense mutations in two genes URA3 and CAN1. In both cases they find that nonsense mutations are unevenly distributed across sites. They find that the factor which explains this distribution best is the curvature of the DNA; more curved sections have lower mutation rates. They show that this pattern is also evident in human cancer genomes; somatic mutations tend to be found in regions with lower curvature. They test whether the pattern is due to curvature by engineering genes in yeast with more or less curvature and showing that increasing curvature decreases the mutation rate. Also, using a repair deficient strain they show that the relationship between curvature and the mutation rate persists, and hence conclude that the correlation between curvature and the mutation rate is not driven by DNA repair, but is due to curved DNA being less accessible to mutagens.

*Response #1*

*We thank Reviewer #2 for the positive evaluation.*

Comment #2

These are interesting results and the analysis and experiments are appropriate. The effect in yeast seems to be stronger than in humans, with curvature seeming to affect the mutation rate by several fold, whereas curvature only affects certain mutational types in cancers, and not as strongly. Do the authors have any explanation for this? Given the rather modest effects in human cancer genomes I wonder whether curvature is a major determinant of the mutation rate in human somatic tissue. Some insight might be gained by performing logistic regression (sites either have a de novo mutation or not) with curvature as one of many variables in the model. I would accept that this might be beyond the scope of the current project.

*Response #2*

*We thank Reviewer #2 for these valuable suggestion. In the revised manuscript we discussed the difference in the impact of DNA curvature on mutation rate between yeast and humans. In yeast, DNA methylation is virtually none and its impact on variation in C>T transition rate is likely small. By contrast, cytosine is extensively methylated in human cells and therefore, the relative importance of intrinsic DNA curvature is likely reduced.*

*We performed logistic regression on cancer mutations to evaluate the relative importance among variables. The impact of DNA curvature on mutation rate can be observed in the logistic regression and its magnitude is similar to an average nucleotide in the 6 flanking sites of mutation site. We added a supplementary figure (Figure S8) showing this result in the revised manuscript.*

Comment #3

Smith et al. (2016 Human Mutation) have found, just as the current authors have, that nonsense mutations are unevenly distributed across sites within human genes. It would be very interesting to see whether this pattern can be explained by curvature.

*Response #3*

*Following this suggestion, we calculated the intrinsic DNA curvature for DNA sequences surrounding the potential nonsense mutation sites in three human genes studied in Smith et al., MECP2, NF1, and RB1. In addition, we included TP53, a tumor suppressor gene that a large number of nonsense mutations have been compiled, into the analysis. We found that intrinsic DNA curvature is negatively associated with the rate of nonsense mutation in all 4 genes. These results further strengthen our conclusions. We added a paragraph in the main text and a supplementary table (Table S3) to present these results in the revised manuscript.*

Comment #4

It would also be interesting to see whether curvature explains some of the variation in the rate of de novo mutation in the human germline; there are now over 140,000 DNMs that have been detected in trios. Again this is probably work for the future.

*Response #4*

*Following this suggestion, we analyzed 101,377 de novo point mutations identified in 1,548 trios from Iceland (Jonsson et al. 2017 Nature). We performed the permutation test as described for mutations in cancer cells (Fig. 3). We found that de novo mutations were significantly enriched in regions with a smaller intrinsic DNA curvature. This observation again strengthens our conclusions. We added a supplementary figure (Figure S9) to present the result in the revised manuscript.*

**Second round of review**

**Reviewer 1:**

The authors have addressed the reviewers' concerns. I am still somewhat doubtful that DNA curvature is really a primary determinant of the observed effect. It might simply be a secondary result of sequence features that are overlooked or that are more difficult to observe. I request that the authors use an established nomenclature for the structural features used (Figure 2a and text). Groove mobility and groove size are not terms used in the nucleic acid community. Instead, groove width and depth are widely accepted structural characteristics. It should furthermore be propeller twist and helix twist (see PMID: 29165643 for a recent paper defining these features). These terms can obviously not be used if the structural features are not calculated in an equivalent way. The manuscript should then clarify the respective relationship and definition. The authors should also discuss their findings in light of another recent paper that connects DNA shape with purifying selection in fly and human (PMID: 29850830).

**Reviewer 2:**

Comments on Duan et al. “Reduced intrinsic DNA curvature leads to increased mutation rate”

This is a very interesting manuscript and the authors have addressed most of my comments satisfactorily. I have a few remaining comments:

•	Although curvature appears to affect the mutation rate in yeast and humans, it may ultimately not be very important in determing variation in the mutation rate if there is little variation in curvature. This is in fact an explanation the authors proffer for why there appears to be an effect of curvature in coding sequences but not the 5’ and 3’ UTR. An alternative explanation here might simply be that the UTRs are shorter, and hence contain less information. Ideally would like to see some quantification how much variation in curvature there is, and what this predicts the variation in the mutation rate to be.

•	The authors refer to mutations in cancer genomes as polymorphisms and SNPs. This is an unconventional use of terminology. I encourage them to use the term single nucleotide variant or somatic mutation to avoid confusing the reader.

It is my standard policy to sign my reviews

Adam Eyre-Walker

**Authors’ response to reviews**

**Point-by-point response to Reviewer #1**

Reviewer #1: The authors have addressed the reviewers' concerns. I am still somewhat doubtful that DNA curvature is really a primary determinant of the observed effect. It might simply be a secondary result of sequence features that are overlooked or that are more difficult to observe. I request that the authors use an established nomenclature for the structural features used (Figure 2a and text). Groove mobility and groove size are not terms used in the nucleic acid community. Instead, groove width and depth are widely accepted structural characteristics. It should furthermore be propeller twist and helix twist (see PMID: 29165643 for a recent paper defining these features). These terms can obviously not be used if the structural features are not calculated in an equivalent way. The manuscript should then clarify the respective relationship and definition.

*We have made these changes accordingly in Figure 2a and text.*

The authors should also discuss their findings in light of another recent paper that connects DNA shape with purifying selection in fly and human (PMID: 29850830).

*We have mentioned that DNA shape is under purifying selection during evolution in the revised manuscript.*

**Point-by-point response to Reviewer #2**

Reviewer #2: Comments on Duan et al. “Reduced intrinsic DNA curvature leads to increased mutation rate”

This is a very interesting manuscript and the authors have addressed most of my comments satisfactorily. I have a few remaining comments:

Although curvature appears to affect the mutation rate in yeast and humans, it may ultimately not be very important in determing variation in the mutation rate if there is little variation in curvature. This is in fact an explanation the authors proffer for why there appears to be an effect of curvature in coding sequences but not the 5’ and 3’ UTR. An alternative explanation here might simply be that the UTRs are shorter, and hence contain less information. Ideally would like to see some quantification how much variation in curvature there is, and what this predicts the variation in the mutation rate to be.

*We have quantified the proportion of the variance in the per-base-pair mutation rate that can be explained by DNA curvature.*

The authors refer to mutations in cancer genomes as polymorphisms and SNPs. This is an unconventional use of terminology. I encourage them to use the term single nucleotide variant or somatic mutation to avoid confusing the reader.

*Corrected.*
